# The Mitochondrial Genome of the Venomous Cone Snail *Conus consors*

**DOI:** 10.1371/journal.pone.0051528

**Published:** 2012-12-07

**Authors:** Age Brauer, Alexander Kurz, Tim Stockwell, Holly Baden-Tillson, Juliana Heidler, Ilka Wittig, Silke Kauferstein, Dietrich Mebs, Reto Stöcklin, Maido Remm

**Affiliations:** 1 Estonian Biocentre, Tartu, Estonia; 2 University of Tartu, Institute of Molecular and Cell Biology, Tartu, Estonia; 3 Institute of Forensic Medicine, University of Frankfurt Medical School, Frankfurt/Main, Germany; 4 J. Craig Venter Institute, Rockville, Maryland, United States of America; 5 Department for Molecular Hematology, University of Frankfurt Medical School, Frankfurt/Main, Germany; 6 Molecular Bioenergetics Group, University of Frankfurt Medical School, Cluster of Excellence Frankfurt Macromolecular Complexes, Frankfurt/Main, Germany; 7 Toxinomics Foundation, Geneva, Switzerland; 8 Atheris Laboratories, Geneva, Switzerland; The City University of New York-Graduate Center, United States of America

## Abstract

Cone snails are venomous predatory marine neogastropods that belong to the species-rich superfamily of the *Conoidea*. So far, the mitochondrial genomes of two cone snail species (*Conus textile* and *Conus borgesi*) have been described, and these feed on snails and worms, respectively. Here, we report the mitochondrial genome sequence of the fish-hunting cone snail *Conus consors* and describe a novel putative control region (CR) which seems to be absent in the mitochondrial DNA (mtDNA) of other cone snail species. This possible CR spans about 700 base pairs (bp) and is located between the genes encoding the transfer RNA for phenylalanine (tRNA-Phe, *trnF*) and cytochrome c oxidase subunit III (*cox3*). The novel putative CR contains several sequence motifs that suggest a role in mitochondrial replication and transcription.

## Introduction

Most metazoan mitochondrial genomes contain 37 genes for 13 protein subunits of the oxidative phosphorylation enzymes, two ribosomal RNAs (rRNAs) and 22 transfer RNAs (tRNAs) [1]. In vertebrates and insects, mitochondrial genomes are well characterized but have been poorly investigated in other taxa. However, studies on molluskan mitochondrial genomes have revealed extensive variations when compared with the mitochondrial genomes of other animals [1], [2], [3], [4]. Among neogastropods, 13 entire mitochondrial genomes have been reported so far [5], [6], [7], [8], [9]. Interestingly, these genomes share a highly conserved gene arrangement with only two cases of tRNA gene translocations [7].

Typically, animal mitochondrial genomes are organized very tightly. They exhibit short intergenic sequences and neighboring genes may even overlap. As an exception, a large non-coding mtDNA region can reach up to several kilobases (kb) in length. In various species, the origin of replication and transcriptional start sites have been identified in this region, leading to the designation of this mtDNA section as the control region (CR) [10].

So far, the mitochondrial genomes of the venomous neogastropods *Conus textile* and *Conus borgesi* have been reported [5], [7]. Cone snails are marine mollusks that produce a complex cocktail of venomous peptides for hunting prey. Because of the specificity and high affinity to a variety of ion channels and receptors, these peptides - also known as conopeptides - have become important tools in pharmacological research and are of considerable interest in drug discovery and development.

Here, we present the mitochondrial genome of the fish-hunting cone snail *Conus consors* and describe some outstanding features of its mitochondrial DNA (mtDNA) sequence in comparison with *C. textile* and *C. borgesi*. This work is part of the Venomics initiative that was launched in 2003 by members of the International Society on Toxinology and joined by the J. Craig Venter Institute (JCVI) in 2005 [11](ref 4). In 2007, as part of CONCO, the Cone Snail Genome Project for Health (see www.conco.eu), a Consortium of CONCO partners was created, with the goal of performing the first genome sequencing of a venomous marine animal and to unravel the complexity of its venomous function.

## Materials and Methods

### Sampling and DNA Extraction

Live *Conus consors* were collected in June 2007 during the CONFIELD-I scientific expedition to the remote Chesterfield Islands in New Caledonia. All necessary permits were obtained for the described field studies from the Official Authorities of New Caledonia. The cone snails were kept in aquariums in the frame of the CONCO project. The foot tissue samples from one specimen of *Conus consors* (sample NC070619AB) were prepared early 2008. Several steps were optimized in order to produce the most DNA of acceptable molecular weight (greater than 10 Kbp dsDNA) per mg of tissue [12]. For Roche/454 sequencing, *Conus consors* genomic DNA (gDNA) was initially isolated using the following steps. DNA extraction was achieved using the EDTA/EGTA/SDS/Proteinase K protocol developed at the J. Craig Venter Institute, which is a modification of standard techniques [13]. 149.8 mg of *Conus consors* foot tissue was ground to powder using mortar and pestle under liquid nitrogen. The pulverized tissue was resuspended in TE/EGTA buffer and cells were lysed by three repeated freeze/thaw cycles, and treated with lysozyme and proteinase K. DNA was extracted with buffer saturated phenol, which was followed by overnight precipitation in 4°C ethanol. DNA extraction was achieved with cetyltrimethylammonium bromide (CTAB) buffer to remove phenolics, polysaccharides, and other PCR inhibitors. The CTAB interface was back-extracted with chloroform to recover additional gDNA, which was followed by phenol, phenol/chloroform extraction, ethanol precipitation and DNA resuspension. It was noted that even after multiple rounds of extraction/precipitation, the *C. consors* DNA exhibited an unusual brown-purple pigment and a relatively high viscosity. For Illumina/Solex sequencing an improved protocol was established for isolating *Conus consors* genomic DNA that was free of any pigments and exhibited a typical viscosity. The protocol was the same as above until DNA precipitation in 4°C ethanol. DNA was then extracted with CTAB/Polyvinylpolypyrrolidone (PVPP) buffer [14] to remove phenolics, polysaccharides, and other PCR inhibitors. DNA was purified on a hydroxyapatite column [15]. Seven column volumes of step gradient of 0.4 M phosphate and 1.0 M phosphate at 60°C were used to elute the gDNA from the column and fractions containing *Conus consors* gDNA were pooled. The *Conus consors* genomic DNA isolated using this revised protocol did not exhibit any pigment and was of typical viscosity. Analysis on 0.8% agarose gel indicated the pooled DNA was larger than 10 Kbp in size. UV/Vis spectrum of the pooled DNA showed a 260 nm/280 nm absorbance ratio of 1.78, and a 260 nm/230 nm absorbance ratio of 1.99. The species was confirmed by PCR using both 16S rRNA and 18S rRNA specific primers as previously described [12].

### Roche/454 Titanium Fragment Library Pyrosequencing

7 µg of high molecular weight *Conus consors* genomic DNA was sheared to 500–800 bp in size using nebulization. Any small fragments generated during nebulization were removed by purification of size-selected DNA from an agarose gel. Size distribution was confirmed using Agilent DNA 7500 chip. A 454 Titanium fragment library was constructed and the quality of the single stranded DNA library was checked by an Agilent RNA Pico 6000 chip. Eighteen full plates of the *Conus consors* 454 Titanium fragment library were run on the Roche/454 Genome Sequencer [16]. The total sequencing generated 17,965,569 reads, and 5,538,744,227 bp of sequence data.

### Illumina/Solexa Sequencing

The *Conus consors* gDNA from the improved isolation protocol was used to construct an Illumina/Solexa 300 bp paired-end library. This library was sequenced on a single lane of an 8 lane flowcell, using a 100 bp paired end read protocol on an Illumina/Solexa GAII sequencer. This single lane produced a total of 56,004,872 reads, and a total of 5,375,369,341 bp of sequence data.

### Genome Assembly

Whole genome assembly was performed using the Roche/454 and Illumina/Solexa data with Roche/454’s GS De Novo Assembler [17]. The GS De Novo Assembler produced 2,091,744 contigs containing 975,801,793 bp of sequence data, had a contig maximum length of 56,875 bp, and a contig N50 value of 610 bp. The genome assembly is highly fragmented due to the abundant low-complexity repeats. Detailed protocol of *Conus consors* genomic DNA assembly and findings from the genome analysis will be presented elsewhere.

### Annotation

The mitochondrial genome sequences of previously sequenced neogastropods were downloaded from NCBI’s RefSeq (http://www.ncbi.nlm.nih.gov/RefSeq/) (see Data S1 for accession numbers). The genomic contigs obtained from *Conus bullatus* were downloaded from the *Conus bullatus* project website http://derringer.genetics.utah.edu/conus/.

Protein coding genes were identified by homology search against available mtDNA sequences from *C. borgesi* [7] and *C. textile* [6]. For tRNA genes two alternative prediction programs were used. *tRNAscan* [18] was run with corresponding settings for organellar tRNA. *ARWEN*, which is designed for predicting metazoan mitochondrial tRNA genes [19], was run with invertebrate-specific settings. In addition, a homology search was performed against the annotated tRNA sequences of *C. textile* and *C. borgesi*. The exact start and end nucleotides of the tRNAs were determined by comparing the results obtained using all three methods. rRNA genes were determined based on homology to corresponding annotations in the mtDNA sequences of *C. textile* and *C. borgesi* which in turn were identified by homology to other mitochondrial rRNA genes. The annotation of 5′ and 3′ ends of 12S rRNA and 16S rRNA sequences is therefore tentative. Potential secondary structures and their folding energies in the control region were predicted using the *mfold* web server (http://mfold.rna.albany.edu/?q=mfold) [20].

### Verification of the Putative Control Region in the mtDNA Sequence of *Conus consors*

The existence of the non-coding putative CR in the mtDNA sequence was verified by semi-manual assembly of the region of interest using additional raw sequencing reads from Illumina/Solexa as described above and previously [12]. The Illumina/Solexa reads were aligned by *MUSCLE* [21] to the automatically assembled mtDNA reference sequence. The resulting alignment was manually verified and tested for assembly errors. No misassembled sections were detected during manual reassembly.

**Figure 1:**
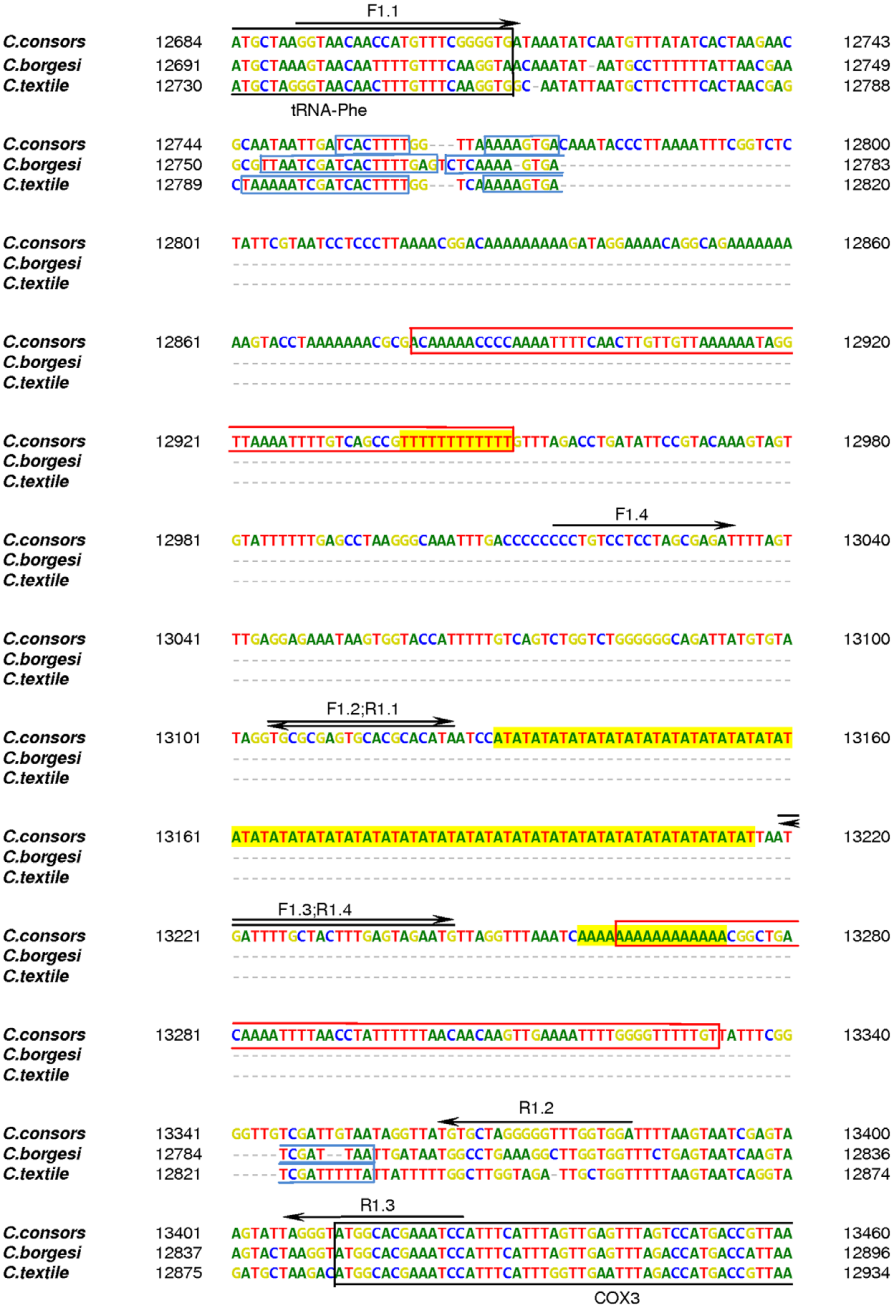
Detailed scheme of the putative mtDNA control region of *Conus consors* in comparison with *C. borgesi* and *C. textile*. The non-coding putative mtDNA control region specific for *C. consors* is located between the genes for tRNA-Phe and COX3. The region spans 698 bp (12713–13411) and exhibits several striking sequence motifs. Comparisons between *C. consors*, *C. borgesi* and *C. textile* show that short inverted repeats (IR1; blue boxes) seem to be a common feature in the mitochondrial genomes of cone snails. However, a long inverted repeat (IR2; red boxes) of 71 bp seems to exist exclusively in *C. consors*. Further, the non-coding putative mtDNA control region of *C. consors* contains several poly(T), poly(A) and poly(AT) sequence motifs (highlighted in yellow). Four primer pairs (F/R1.1 - F/R1.4) were employed to validate the exact length of the poly(T), poly(A) and poly(AT) motifs and are shown with black-arrowed lines (*F* - forward, *R*- reverse).

In addition to the second-generation sequencing strategies, a PCR-based approached followed by classical Sanger sequencing was employed to verify and to determine the exact lengths of several sequence motifs (poly(A), poly(T) and poly(AT)) in the putative mtDNA CR of *C. consors*. According to the assembly of the mitochondrial genome derived from the Illumina/Solexa data, Primer3 (http://biotools.umassmed.edu/bioapps/primer3_www.cgi) was utilized to generate four primer pairs flanking the sequence motifs of interest (see Figure 1; Forward (F)1.1: GGTAACAACCATGTTTCGGGGTGA and Reverse (R)1.1: TGCGCGAGTGCACGCACATA, PCR protocol: 94°C for 10 min – (94°C for 45 sec –65°C for 45 sec –68°C for 45 sec) x40 cycles –68°C for 10 min, PCR product: 434 bp; F1.2: TGCGCGAGTGCACGCACATA and R1.2: TGTGCTAGGGGGTTTGGTGGA, PCR protocol: 94°C for 10 min – (94°C for 45 sec –56°C for 45 sec –68°C for 45 sec) x40 cycles –68°C for 10 min, PCR product: 279 bp; F1.3: TGATTTTGCTACTTTGAGTAGAATG and R1.3: TAGGGTATGGCACGAAATCC, PCR protocol: 94°C for 10 min – (94°C for 45 sec –52°C for 45 sec –68°C for 45 sec) x40 cycles –68°C for 10 min, PCR product: 206 bp; F1.4: CCCTGTCCTCCTAGCGAGAT and R1.4: TGATTTTGCTACTTTGAGTAGAATG, PCR protocol: 94°C for 10 min – (94°C for 45 sec –52°C for 45 sec –68°C for 45 sec) x40 cycles –68°C for 10 min, PCR product: 230 bp). Each PCR (total volume of 25 µl) contained 1.5 U AmpliTaq-Gold® polymerase (Applied Biosystems; Foster City, CA/USA), 4 µmol dNTPs (GE Healthcare Europe; Freiburg, Germany), 8 ng bovine serum albumin (BSA – Sigma-Aldrich; Taufkirchen, Germany), 10 pmol forward-primer, 10 pmol reverse-primer and 5 ng of total DNA obtained from *C. consors*. Amplified PCR products were separated via agarose gel electrophoresis. Those PCR products exhibiting the expected (or an approximate) size were excised and purified using the QIAquick® Gel Extraction Kit (QIAGEN; Hilden, Germany). Extracted PCR products were ligated into a pGEM®-T expression vector (pGEM®-T Vector System II, Promega; Madison, WI/USA). JM109 competent cells (Promega; Madison, WI/USA) were transformed with the corresponding vector constructs. Successfully transformed clones were selected, amplified and the plasmid constructs extracted using the QIAprep® Spin Miniprep Kit (QIAGEN; Hilden, Germany). Prior to Sanger sequencing, the corresponding *C. consors*-derived inserts were reamplified via PCR by utilizing the vector-specific primers T7 (TAATACGACTCACTATAGGG) and SP6 (ATTTAGGTGACACTATAGAA). These primers were also employed as sequencing primers. Sequence analysis was performed using the BigDye® Terminator v3.1 Cycle Sequencing Kit (Applied Biosystems) according to the manufacturer’s instructions. Amplified DNA samples were purified by utilizing the DyeEx® 2.0 Spin Kit (QIAGEN) according to the manufacturer’s instructions, denatured for 2 min at 95°C and sequenced on an ABI 3130 Genetic Analyzer (Applied Biosystems). Obtained DNA sequences specific for the mtDNA of *C. consors* were analyzed using *Sequencing Analysis v5.2* (Applied Biosystems) and aligned via *Clustal W2* [22].

## Results

### Gene Content and Arrangement of the Mitochondrial Genome of *C. consors*

**Figure 2:**
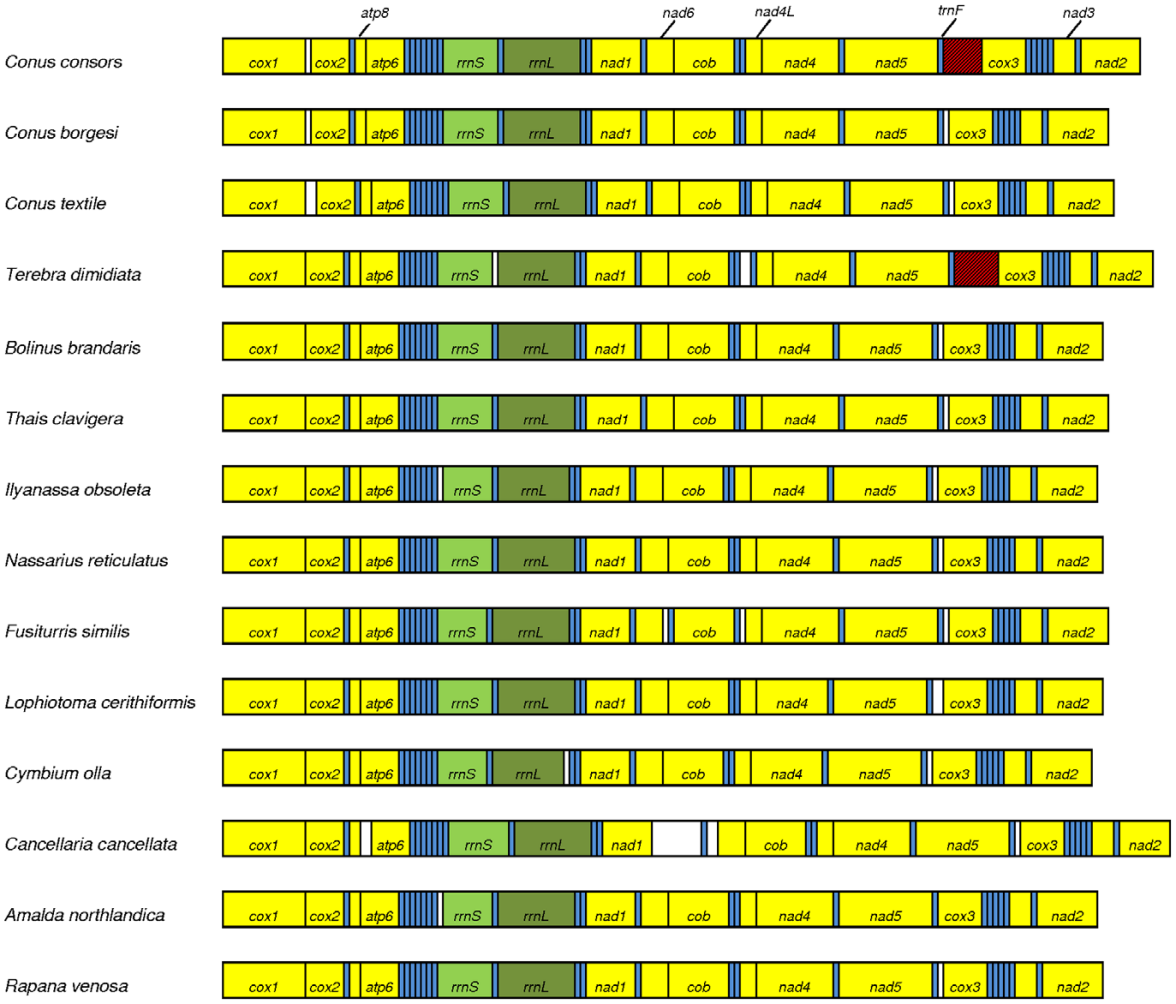
Gene arrangement of the mitochondrial genome of *Conus consors* in comparison with other neogastropods. The gene order of the mitochondrial genome of *C. consors* was compared with the mitochondrial genomes of two other cone snails (*C. borgesi* and *C. textile*) and further marine neogastropods. The scheme visualizes genes encoding proteins (yellow), transfer-RNAs (blue) and ribosomal RNAs (green). Non-coding mtDNA regions larger than 50 bp are white-colored, while the non-coding and putative control region between the genes for tRNA-Phe and COX3 in the mitochondrial genomes of *C. consors* and *T. dimidiata* is highlighted in red.

The mtDNA of *C. consors* was sequenced and assembled using Illumina/Solexa and Roche/454 second-generation sequencing platforms. The assembled mtDNA sequence was 16,112 bp in size. The heavy strand of the mtDNA molecule consisted of 27.7% adenine (A), 13.5% cytosine (C), 19.4% guanine (G) and 39.4% thymine (T) with a total (A/T) content of 67.1%. The mitochondrial genome contained 13 genes encoding proteins of the respiratory chain, 22 tRNA genes and 2 rRNA genes. All protein coding genes as well as the tRNAs for Asp, Val, Leu1-2, Pro, Ser1-2, His, Phe, Lys, Ile, Ala, Arg, Asn and both ribosomal RNA genes were located on the heavy strand of the mtDNA molecule. In contrast, the tRNAs for Met, Tyr, Cys, Trp, Gln, Gly, Glu and Thr were located on the light mtDNA strand. All protein coding genes commenced with the standard ATG start codon. The gene order of the mitochondrial genome of *C. consors* was similar to that of other neogastropods for which sequence data are available [5], [6], [7], [8], [9] (Figure 2). Remarkably, sequence overlaps existed between the same genes and showed exactly the same length as in *C. textile* [6]. The longest overlap (7 bp) was observed for the genes encoding NADH dehydrogenase subunit 4 L (*nad4L*) and NADH dehydrogenase subunit 4 (*nad4*).

### The Longest Intergenic mtDNA Sequence Exhibits Several Characteristics of a Control Region

The longest intergenic sequence in the mitochondrial genome of *C. consors* was found to be located between the genes for tRNA-Phe (*trnF*) and cytochrome c oxidase subunit III (*cox3*). The size of this region was 698 bp, five-fold longer than in the mtDNA of *C. textile* and *C. borgesi* with 126 bp and 127 bp, respectively [5], [7]. In animal mitochondrial genomes, the longest intergenic part of the mtDNA sequence usually contains several elements which regulate the initiation of replication and transcription and is therefore referred to as control region (CR) [1]. Based on analogy in the mitochondrial genomes of *Lophiotoma cerithiformis* and *Ilyanassa obsoleta*, the intergenic sequence between *trnF* and *cox3* was suggested to represent the CR in the mitochondrial genome of *C. textile* [6]. Indeed, in *C. consors*, the mtDNA region between *trnF* and *cox3* showed several CR-specific characteristics: (I) the sequence represented the longest non-coding region in the mitochondrial genome, (II) exhibited a high (A/T) content (70.3%) and (III) contained possible stem-loop-like secondary structures as well as (IV) repetitive elements.

Interestingly, numerous homopolymeric sequence motifs (poly(A), poly(T) and a long poly(AT) tandem repeat stretch) were detected in the putative mtDNA CR of *C. consors*, while no comparable sequence motifs were reported in the mitochondrial genomes of *C. textile* and *C. borgesi*. In these cone snail species, the corresponding intergenic mtDNA sequence was found to be more than five times shorter than in *C. consors*. Recently, a partial nuclear genome of another cone snail (*Conus bullatus*) has been reported [23]. None of the *C. bullatus* contigs provided matches against the putative mtDNA CR detected in the cone snails *C. consors*, *C. borgesi* and *C. textile*. Based on homology, some short contigs belong to the mitochondrial genome but no full-length sequence is available. Therefore, the existence of a similar CR on the mitochondrial genome of *C. bullatus* is currently unclear.

In order to exclude potential artifacts due to sequencing and/or data assembling repeated regions in the control region, the corresponding mtDNA section was further investigated. The original assembly which was based on Roche/454 sequencing reads contained 44 (AT) tandem repeat motifs (poly(AT)_44_) in the putative CR. Additional reads obtained from Illumina/Solexa sequencing were not useful to estimate the exact length of the poly(AT) stretch. These reads either ended before the poly(AT) sequence motif or reached up to a maximum of seven (AT) tandem repeats (poly(AT)_7_) only (data not shown). However, the other parts of the CR were well covered and confirmed the existence of long intergenic sequence motifs in the mitochondrial genome of *C. consors*. In addition, homology studies indicated that the CR did not exhibit any sequence homology in the nuclear genome of *C. consors* (data not shown). Thereby the possibility that sequencing reads from the nuclear genome had been misassembled into the mitochondrial genome was excluded.

The putative CR between *trnF* and *cox3* in the mitochondrial genome of *C. consors* represents the longest non-coding mtDNA sequence known among the cone snail species investigated so far (Figure 2). Although differing in length, this mtDNA region in *C. consors*, *C. borgesi* and *C. textile* is sharing one common aspect: All three mitochondrial genomes contain a short inverted repeat (IR1) motif that is very similar regarding sequence and location (Figure 1). In *C. textile* and *C. borgesi*, the IR1 contains 18 bp and 19 bp, respectively. Here, in *C. consors*, the IR1 was found to be shorter (8 bp) and immediately followed by a long segment, which is missing in the mtDNA of the other two cone snail species.

Furthermore, another, even longer inverted repeat (IR2) sequence motif consisting of more than 70 bp and surrounding the stretch of poly(AT) tandem repeats (Figure 1) was found in the mitochondrial genome of *C. consors*. However, the exact length of the IR2 region remained unclear due to the existence of poly(T) and poly(A) sequence motifs. Based on the original assembly, the IR2 contained poly(T)_12_ and poly(A)_16_ homopolymers. Interestingly, manual validation with additional Illumina/Solexa sequencing reads resulted in longer homopolymer tracts, poly(T)_22_ and poly(A)_22_, respectively.

In order to verify and to determine the exact length of the poly(T) and poly(A) IR2 sequence motifs, the putative CR was also analyzed in an additional and independent PCR-based approach followed by classical Sanger sequencing. Since poly(AT) motifs have been reported to create problems in CR sequencing [24], [25], this experimental strategy was also employed to determine the exact length of the poly(AT) stretch. Optimized PCR protocols were developed and applied to amplify the IR2 sequence parts containing the poly(T) and poly(A) motifs as well as the poly(AT) stretch. PCR-based amplification of both IR2 sequence parts including the poly(T) and poly(A) motifs was successful. However, in case of the poly(AT) stretch, no distinct PCR products of the expected size were obtained. PCR products exhibiting the expected size (poly(T) and poly(A) analyses) or an approximate size (poly(AT) analyses) were employed for Sanger sequencing.

**Figure 3:**
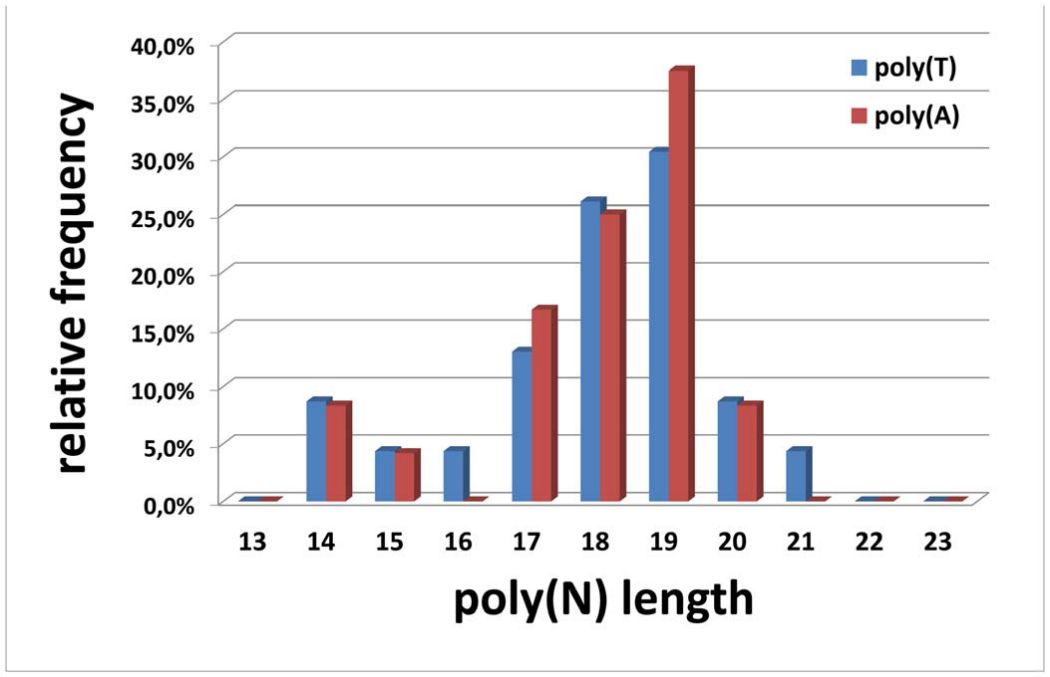
Analyses of poly(T) and poly(A) motifs in the IR2 sequence of *Conus consors*. 25 clones representing the first IR2 sequence part (blue bars; primer pair F/R-1.1, PCR product includes poly(T) stretch) and 17 clones representing the second IR2 sequence part (red bars; primer pair F/R-1.3, PCR product includes poly(A) stretch) were sequenced according to Sanger and analyzed. Since, the quality of the poly(A) sequences obtained had been suboptimal, the reverse complement sequences containing a poly(T) stretch instead of the poly(A) motif were analyzed. For both IR2 sequence parts investigated, variations in length due to the phenomenon of strand slippage were observed. Accordingly, it was not possible to determine the exact length of the poly(T) and the poly(A) stretches. Most likely, both homopolymeric sequence motifs are 18 or 19 bp in length.

Sequence analyses of multiple clones confirmed the existence of both IR2 poly(T) and poly(A) sequence motifs as well as the presence of poly(AT) tandem repeats. The length of the homopolymeric poly(T) and poly(A) sequence motifs and the poly(AT) tandem repeat stretch could not exactly be determined. However, the data obtained by Sanger sequencing suggested that, most likely, both homopolymeric IR2 sequence motifs (poly(T) and poly(A)) are 18 or 19 bp in length (Figure 3).

In order to determine the exact length of the poly(AT) tandem repeat stretch and to exclude errors of the sequencing data by contamination with genomic DNA, a novel protocol for the extraction of mtDNA from cone snail tissue was established and pure mtDNA was employed for Sanger sequencing (see Data S1, S2, S3). The existence of the poly(AT) sequence motif was confirmed by this experimental strategy. However, the exact length of the stretch was still not clear, since the detected signals could not be analyzed after 10 to 12 poly(AT) tandem repeats (data not shown).

**Figure 4:**
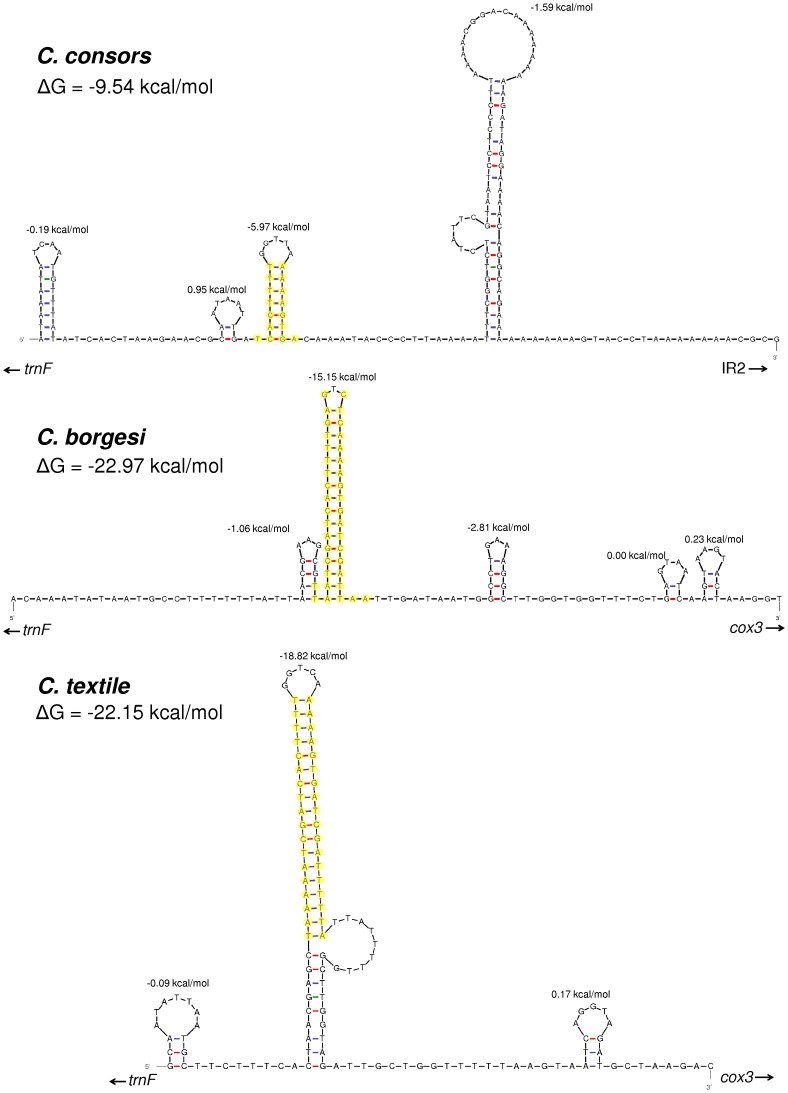
Predicted secondary structures before IR2 of the mtDNA control region of *Conus consors* in comparison with *C. borgesi* and *C. textile*. Short inverted repeat IR1 (highlighted with yellow circles) is a common feature of the non-coding region in the mitochondrial genomes of *C. consors*, *C. borgesi* and *C. textile*. Structures and folding energies were predicted with *mfold* [20].

In invertebrates the CR apparently lacks conserved sequence blocks and is not as well characterized as in vertebrates. However, it is known that in *Drosophila* mtDNA the origin of the light/minor coding strand is located in the middle of the large AT-rich non-coding region [26]. Increased AT-content is one characteristic that is used for the identification of the replication origins. Similarly, the AT-rich section in *C. consors* mtDNA could refer to the existence of the replication origin inside the intergenic sequence between the genes for *trnF* and *cox3*. Among vertebrate species, the conserved sequence located around the replication origin of the light strand could form stem-loop configuration which is suggested to be required for the initiation of replication by acting as the recognition structure for the mitochondrial primase enzyme [27]. The analysis of the secondary structure of the mtDNA molecule via *mfold* showed that the region preceding the IR2 has a potential to form stem-loop-like structures (Figure 4). Interestingly, one of these structures is formed by the IR1 and also found in the mtDNA of *C. textile* and *C. borgesi* (Figure 1, 4). There exists also a larger but weaker stem-loop after IR1 (Figure 4). The largest and *C. consors* specific inverted repeat IR2 could also form a complex secondary structure (Figure S1). It is unclear whether such a complex structure actually exists and exhibits a regulatory function.

**Figure 5:**
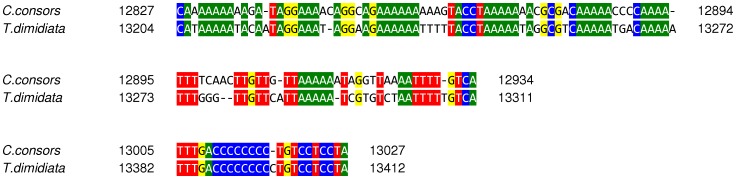
Highly similar segments of the longest non-coding mtDNA region in the neogastropods *Conus consors* and *Terebra dimidiata* [**7**] **.** CRs in *C. consors* and *T. dimidiata* share short and very similar segments, including the 39 bp of the IR2 sequence motif (the second alignment). Sequence alignment was done by MUSCLE [20]. Sequence similarities are highlighted in green (A), blue (C), yellow (G) and red (T).

## Discussion

To date, 13 complete mitochondrial genomes of snails from the order of the Neogastropoda are available and show a very high level of similarity [5], [6], [7], [8], [9]. Among cone snails, the order and even the overlaps of the mitochondrial genes are highly conserved. It is interesting to note that mtDNA sequences of cone snails show a major difference regarding the putative CR between *trnF* and *cox3*. Rather, the CR of the fish-hunting species *C. consors* shares several similar motifs with the phylogenetically more distant *Terebra dimidiata*, a marine snail from the family of the Terebridae [28], [29]. So far, *T. dimidiata* [7], [28], [29], another venomous marine snail feeding on worms, is the only known neogastropod which exhibits a similar non-coding and putative mtDNA CR between *trnF* and *cox3* observed in *C. consors*. However, this region is even longer (848 bp) and does not align well with the putative CR in the mitochondrial genome of *C. consors*. Both regions share short and very similar segments, including the preceding mtDNA sequence and also 39 bp of the IR2 sequence motif itself (Figure 5). It would be of interest to investigate the possible correlation of this observation with the venomous function. So far, no long IRs in the CR of the mitochondrial genome of *T. dimidiata* have been reported. However, in that genome, a short (10 bp) IR is located in the mtDNA region which is homologous to the IR2 sequence motif of *C. consors*. In addition, the intergenic mtDNA sequence between *trnF* and *cox3* in *T. dimidiata* contains a poly(AT) motif, but it is shorter and preceded by a poly(AC) tandem repeat. No similar mtDNA segments were observed between the putative control region of *C. consors* and the largest intergenic sequence of *Cancellaria cancellata*, located between the genes for NADH dehydrogenase subunit 1 (*nad1*) and the tRNA for proline (*trnP*). The CR is found to be the fastest evolving region of the mtDNA [30], [31]. It is hard to speculate which specific mechanisms are involved in the CR variation among cone snails. In some organisms long non-coding regions were shown to contain pseudogenes [32], [33]. However, no known pseudogenes were observed in the mtDNA of *C. consors*. When comparing the intergenic mtDNA region between *trnF* and *cox3* with the whole mitochondrial genome sequence, no homologies were found that would refer to duplications of the coding areas. Also, no homologies were detected when compared to the corresponding nuclear genome sequence. A comparative study of the mitochondrial genomes from other cone snail species and neogastropods would be useful to estimate the extent of variation of the long non-coding mtDNA sequences in this group. It is likely that the variable control region will prove to be additional feasible and valuable marker sequence for phylogenetic analysis of cone snails or neogastropods. The existence of comparable region in *T. dimidiata* supports the potential usability, however due to the limited number of available mitochondrial genome sequences among neogastropods it remains to be studied in the future.

Tandem repeats (like poly(AT) motifs) as well as the high (A/T) content are well known features of the CRs in animal mtDNA [1]. Poly(AT) stretches have been described in several putative CRs of various species including mollusks [34], [35], [36], [37], [38]. The present data suggests that the mtDNA CR of *C. consors* exhibits 44 poly(AT) tandem repeats. Notably, poly(AT) stretches of a similar or larger size have been identified in non-coding regions of other molluskan mitochondrial genomes as well, e.g. in *Haliotis rubra* [25] and *Katharina tunicata* [34].

The inconsistencies observed regarding the lengths of the poly(T) and poly(A), as well as the poly(AT) sequence motifs in the mtDNA CR of *C. consors* may be due to sequencing and/or assembling errors. It is also possible that those mtDNA regions could be highly polymorphic. Previously, several variations of the poly(AT) stretches in molluskan mitochondrial genomes have been reported [39], [40], [41]. Therefore, it would be reasonable to compare the lengths of the poly(AT) stretches in other specimens of *C. consors* to prove whether such polymorphisms also occur in this cone snail species. On the other hand, several variations of the mtDNA region may exist due to a phenomenon called strand slippage which can occur during enzymatic replication. Strand slippage is a result of mispairing between the template and the newly synthesized strand and can occur especially when long homopolymeric and/or short tandem repeat sequence motifs are present.

Several sequence motifs similar to well known *cis*-acting elements are also present in the mtDNA CR of *C. consors*. The ATATAA box is a common modular element for most promoters. The motif TATATATAA as a consensus sequence has been identified in the sea urchin *Arbacia lixula* [42] as well as the marine mussel *Mytilus* [43] and may represent a bidirectional promoter [44] or a binding site for transcription termination factors [44], [45]. It is possible that the mtDNA sequence region around the poly(AT) stretch in *C. consors* exhibits a similar function. The conserved sequence motif GYRCAT is present in the terminal associated sequences (TAS) of mammalian and avian mitochondrial CRs [46], [47]. The TAS element is considered to represent the termination site of the mtDNA’s heavy strand synthesis [48]. A similar sequence motif, GCACAT, precedes the poly(AT) stretch in *C. consors*. However, it is difficult to prove whether these sequence motifs play a role in the regulation of mtDNA homeostasis in *C. consors*.

The sequence of the second largest intergenic region in *C. consors*, located between the genes *cox1* and *cox2*, has been previously published [6]. The alignment of this 132 bp long sequence with the corresponding region in the current assembly revealed nine mismatches (6.8%). All of those nine mismatches were transition type changes (purine-purine and pyrimidine-pyrimidine). Whether these differences represent the actual polymorphisms remains to be verified.

### Conclusions

Here, we present the sequence and gene annotation of the mitochondrial genome of the cone snail *Conus consors*. The mitochondrial genome contains a long intergenic sequence (about 700 bp) that exhibits several characteristic motifs typical for the control region of animal mtDNA. Remarkably, the reported putative CR is unique among the mitochondrial genomes of cone snails since a comparable mtDNA region has not been described for other *Conus* species investigated so far.

## Supporting Information

Figure S1 ***mfold*** **predicted secondary structure for the whole CR of** ***Conus consors*** **.** IR2 could potentially form a large stem-loop-like structure.
(PDF)

Data S1 **Accession numbers of Neogastropoda mitochondrial genomes.**
(PDF)

Data S2 **Preparation of mitochondria from fresh or frozen** ***Conus consors*** **tissue samples.**
(PDF)

Data S3 **Isolation of mitochondrial DNA.**
(PDF)
