# Supplementary material for: The Mitochondrial Genome of the Venomous Cone Snail Conus consors
Source: PLoS One. 2012 Dec 7;7(12):e51528. doi: 10.1371/journal.pone.0051528 (PMC3517553; doi:10.1371/journal.pone.0051528)
Supplement: Figure S1 — mfold predicted secondary structure for the whole CR of Conus consors . IR2 could potentially form a large stem-loop-like structure. (PDF) [file pone.0051528.s001.pdf]

Supplemental Figure S1

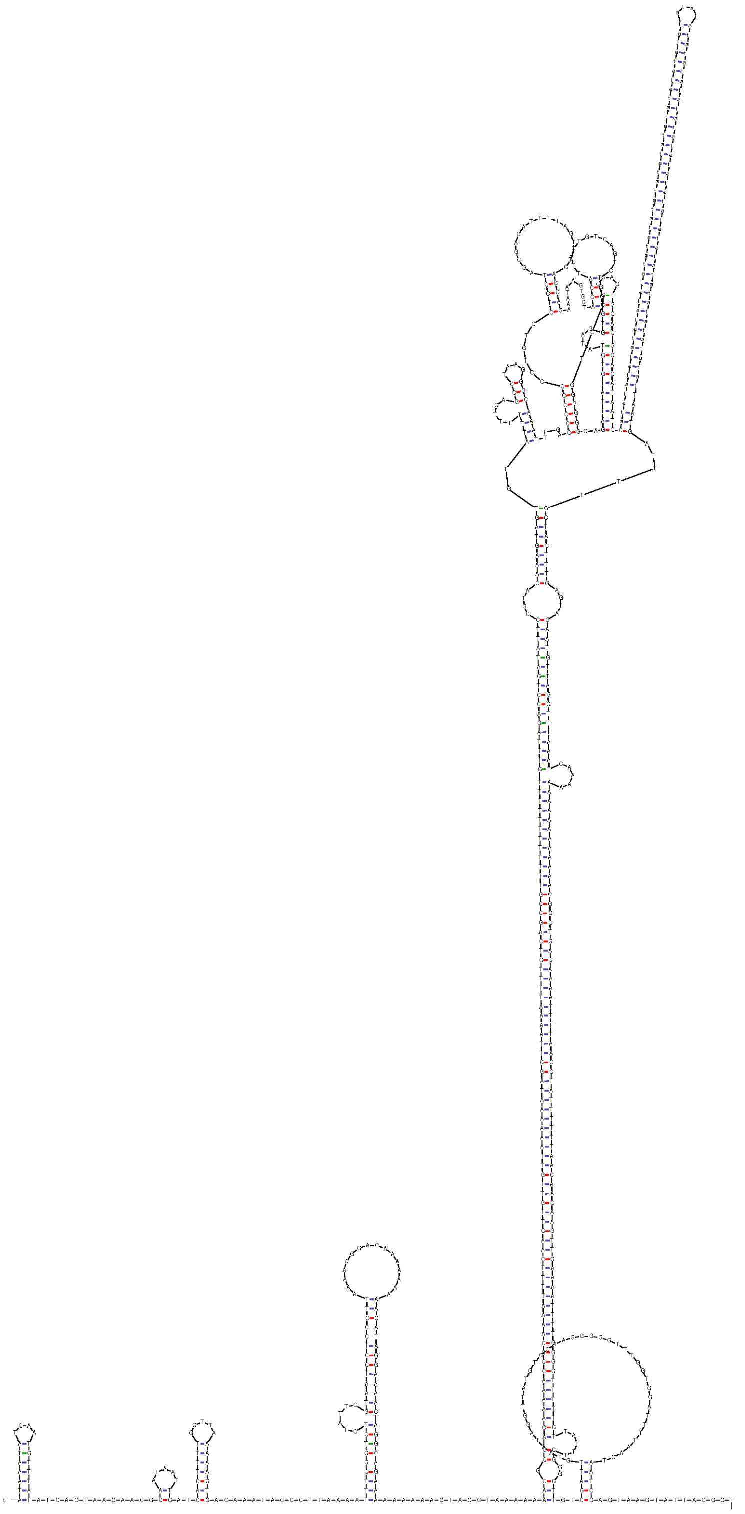

Figure S1: *mfold* predicted secondary structure for the whole CR of *Conus consors*. IR2 could potentially form a large stem-loop-like structure.
