# Supplementary material for: The Mitochondrial Genome of the Venomous Cone Snail Conus consors
Source: PLoS One. 2012 Dec 7;7(12):e51528. doi: 10.1371/journal.pone.0051528 (PMC3517553; doi:10.1371/journal.pone.0051528)
Supplement: Data S1 — Accession numbers of Neogastropoda mitochondrial genomes. (PDF) [file pone.0051528.s002.pdf]

**Accession numbers of Neogastropoda mitochondrial genomes**

*Conus borgesi* NC_013243.1, *Conus textile* NC_008797.1, *Terebra dimidiata* NC_013239.1, *Bolinus brandaris* NC_013250.1, *Thais clavigera* NC_010090.1,  Ilyanassa obsoleta NC_007781.1, *Nassarius reticulatus* NC_013248.1, *Fusiturris similis* NC_013242.1, *Lophiotoma cerithiformis* NC_008098.1, *Cymbium olla* NC_013245.1, *Cancellaria cancellata* NC_013241.1, *Amalda northlandica* NC_014403.1, *Rapana venosa* NC_011193.1.
