# Supplementary material for: The Mitochondrial Genome of the Venomous Cone Snail Conus consors
Source: PLoS One. 2012 Dec 7;7(12):e51528. doi: 10.1371/journal.pone.0051528 (PMC3517553; doi:10.1371/journal.pone.0051528)
Supplement: Data S2 — Preparation of mitochondria from fresh or frozen Conus consors tissue samples. (PDF) [file pone.0051528.s003.pdf]

Preparation of mitochondria from fresh or frozen *Conus consors* tissue samples

In brief, mtDNA was extracted from the mitochondrial fraction derived from fresh and/or frozen foot tissue of several *C. consors* specimens. With the aim to amplify the poly(AT) stretch, the pure mtDNA was employed in the already described PCR-based approach followed by Sanger sequencing.

Cone snail tissues were prepared from foot and/or liver and stored at -80°C in RNA later (QIAGEN) or ethanol. Prior use, the samples conserved in ethanol were washed five times in TE buffer (10 mM Tris (pH 8.0), 1 mM EDTA) for 1 h at room temperature followed by a final incubation over night at 4°C.
The tissues were rinsed in ice-cooled buffer (250 mM sucrose, 10 mM Tris (pH 7.5), 1mM EDTA, 5 mM aminocaproic acid), cut into small pieces and homogenized using a glass/Teflon Potter-Elvehjem homogenizer (1,500 rpm, 30 strokes). Homogenates were centrifuged for 5 min at 4°C and 600 g. Mitochondrial membranes were sedimented by centrifugation of the supernatants for 15 min at 4°C and 8,000 g. Isolated mitochondrial DNA was purified by agarose gel extraction (Machery-Nagel; Düren, Germany).
