# Supplementary material for: The Mitochondrial Genome of the Venomous Cone Snail Conus consors
Source: PLoS One. 2012 Dec 7;7(12):e51528. doi: 10.1371/journal.pone.0051528 (PMC3517553; doi:10.1371/journal.pone.0051528)
Supplement: Data S3 — Isolation of mitochondrial DNA. (PDF) [file pone.0051528.s004.pdf]

Isolation of mitochondrial DNA

Mitochondrial pellets were lysed in TENS buffer (50 mM Tris/HCl (pH 8.0), 40 mM EDTA (pH 8.0), 100 mM NaCl, 1 % SDS) and incubated with RNase A (200 µg) at 37 °C and Proteinase K (200 µg) at 56 °C for 30 min each. The mitochondrial DNA was extracted by phenol:chloroform:isoamyl alcohol (25:24:1) and subsequently precipitated by isopropanol. Finally, the mtDNA pellets were dissolved in TE buffer (10 mM Tris (pH 8.0), 1 mM EDTA).

All chemicals were obtained from Sigma-Aldrich (Taufkirchen, Germany). RNase A and Proteinase K were obtained from Fermentas (St. Leon-Roth, Germany).
